# A Molecular Epidemiological Survey of Tick-Borne Pathogens in Dogs and Their Associated Ticks in Xinjiang, China

**DOI:** 10.3390/ani16040534

**Published:** 2026-02-08

**Authors:** Yongchang Li, Jiaxin Li, Jianlong Li, Yang Yang, Fakiha Kalim, Iqra Zafar, Bayin Chahan, Qingyong Guo

**Affiliations:** 1 College of Veterinary Medicine, Xinjiang Agricultural University, Urumqi 830052, China; yongchangli@xjau.edu.cn (Y.L.); 13999249170@163.com (J.L.); xnddyljl@xjau.edu.cn (J.L.); bayinchahan@xjau.edu.cn (B.C.); 2 National Research Center for Protozoan Diseases, Obihiro University of Agriculture and Veterinary Medicine, Obihiro 080-8555, Japan; 3 Laboratory of Sustainable Animal Environment, Graduate School of Agriculture Science, Tohoku University, Sendai 980-8577, Japan

**Keywords:** canine, Xinjiang, tick-borne pathogens (TBPs)

## Abstract

Tick-borne diseases (TBDs) pose a significant global threat to both canine and public health, largely attributable to the extensive geographic distribution of ticks and their ability to harbor diverse pathogens. To evaluate regional risk, this study examined the developmental biology of a prevalent tick species in Xinjiang, China, and performed molecular surveillance for key pathogens in both tick vectors and canine hosts. Observations of reproductive biology revealed that *Riphicephalus turanicus* ticks could complete their development from egg to adult in approximately 50 days on laboratory rabbits, indicating a rapid lifecycle and high reproductive potential. Polymerase chain reaction (PCR)-based screening of 379 canine blood samples detected infection rates of 14.25% for *Anaplasma* spp., 2.64% for *Hepatozoon* spp., 21.64% for *Rickettsia* spp., and 21.90% for *Babesia* spp. Concurrently, screening of 184 tick samples revealed detection rates of 15.22% for *Anaplasma ovis*, 8.15% for *Hepatozoon* spp., and 21.74% for *Rickettsia* spp. Statistical analysis identified significant regional variation in pathogen prevalence across the surveyed locations. The BLASTn (BLAST: Basic Local Alignment Search Tool) alignment revealed high sequence identity (99.30–100%) with known strains of *Babesia*, *Rickettsia*, and *Anaplasma* circulating in Asia and Europe. confirming the presence of these pathogens in the local ecosystem and evolutionary linkage to global lineages. Collectively, these findings provide valuable epidemiological insight into the endemic nature of TBDs in Xinjiang and emphasize the importance of integrated tick management and sustained disease surveillance programs.

## Simple Summary

Ticks represent a significant public and veterinary health challenge due to their capacity to transmit serious pathogens to canines and humans. This study aimed to characterize the threat posed by ticks in Xinjiang, China, by addressing two primary objectives: determining the developmental timeline of local tick species and identifying the associated tick-borne pathogens. Our findings revealed a rapid developmental cycle from egg to adult of approximately 50 days, indicating a high reproductive capacity. Furthermore, screening of canine blood and tick samples detected high infection rates of *Anaplasma* spp., *Babesia* spp., and *Rickettsia* spp. Genetic analysis confirmed that the detected pathogen sequences share high sequence identity with strains circulating worldwide. This research provides essential epidemiological evidence that underscores the urgent need for robust tick-control strategies and raises awareness of the risk posed by endemic tick-borne diseases in the region.

## 1. Introduction

Tick-borne diseases constitute a major group of vector-borne illnesses worldwide. Through hematophagous feeding, ticks can transmit a wide range of pathogens to both humans and animals, including Crimean-Congo hemorrhagic fever, babesiosis, anaplasmosis, Lyme disease, ehrlichiosis, and rickettsiosis [1]. Although canines and humans serve as common hosts and share epidemiological risks, targeted research and clinical documentation in this field remain limited.

In China, over 117 tick species across seven genera and more than 30 emerging tick-borne pathogens (TBPs) have been documented. The Xinjiang Uygur Autonomous Region (XUAR) encompasses one-sixth of China’s territory and borders eight countries, has documented the presence of six tick genera including *Dermacentor*, *Hyalomma*, *Rhipicephalus*, *Haemaphysalis*, *Ixodes*, and *Argas*, comprising 14 identified species [2]. Epidemiological surveys have identified *Rhipicephalus turanicus*, *Dermacentor niveus*, *Hyalomma asiaticum*, and *Dermacentor marginatus* as the predominant species in this region [2].

Among the pathogens impacting canine health, *Hepatozoon canis*, *Babesia canis*, *Rickettsia* spp., and *Anaplasma* spp. are of growing concern, as they are associated with clinical manifestations such as anemia, pyrexia, and mortality. The brown dog tick, *Rhipicephalus sanguineus* (*Rh. sanguineus*) is recognized as a major global vector for *H. canis* [3]. Notably, *H. canis* has been detected in territories adjacent to Xinjiang, such as Pakistan [4] and Afghanistan [5]. Small mammals serve as primary reservoirs for *Babesia* spp. and *Hepatozoon* spp., while canine infections frequently resulting from infected invertebrate hosts [5]. *Babesia* spp. infections in dogs, particularly those caused by *B. gibsoni*, *B. vogeli*, and *B. canis*, have been reported in neighboring countries like India [6], Kazakhstan [7], and Kyrgyzstan [8]. These pathogens have also been isolated from various tick species, including *Haemaphysalis longicornis*, *Hyalomma aegyptium*, *H. anatolicum*, *Haemaphysalis sulcata*, *Ixodes persulcatus*, *Rh. turanicus*, *Rh. microplus*, and *Rh. sanguineus*, across Russia [9], Kyrgyzstan [10], and Pakistan [11].

Globally, the canine population is estimated at approximately 900 million, comprising both owned and stray dogs [12]. Stray dogs constitute nearly 75% of this demographic, particularly in regions with underdeveloped veterinary services and limited pet regulation systems. According to industry reports such as the China Pet Industry White Paper, urban areas of China were home to roughly 54 million companion dogs as of 2023.

As one of the most ubiquitous companion animals worldwide, dogs also fulfill specialized roles, such as service animals for the visually impaired and search-and-rescue units, further integrating them into human societal frameworks. Despite their pivotal role as both household companions and potential sentinels for zoonotic risk, comprehensive data on the prevalence and heterogeneity of tick-borne pathogens in these populations, particularly in high-risk frontier regions like Xinjiang remain scarce. This dearth of surveillance impedes effective risk assessment and the development of targeted control strategies. Therefore, the present study aims to delineate the current molecular landscape of major tick-borne pathogens circulating in domestic and stray dogs in Xinjiang, thereby providing critical data for informed public health and veterinary interventions.

## 2. Materials and Methods

### 2.1. Samples and Reagents

**Figure 1:**
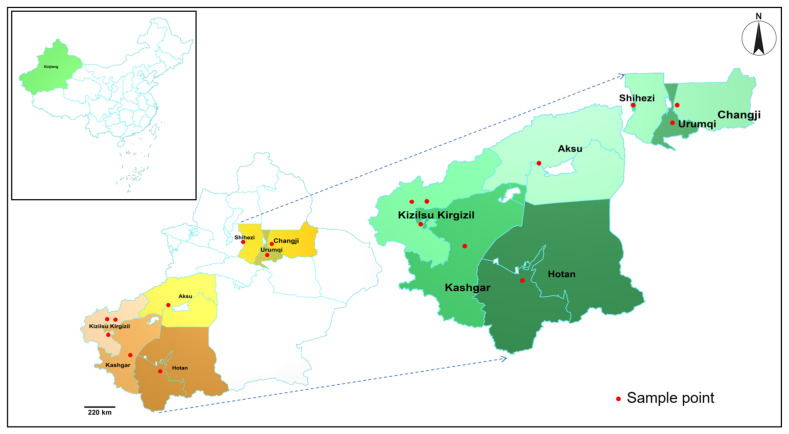
Map indicating the sampling locations in selected areas of Xinjiang.

This study was conducted between March 2024 and September 2025, encompassing the spring, summer, and early autumn seasons in Xinjiang, which corresponds to the period of peak tick activity. Canine blood samples were obtained from dogs presented to the Animal Hospital of Xinjiang Agricultural University. A total of 379 canine blood samples were collected from dogs across northern and southern regions of Xinjiang, including the cities and prefectures of Urumqi, Changji, Shihezi, Bole, Hotan, Kashgar, Kizilsu Kyrgyz Autonomous Prefecture, Wujiaqu and Aksu (Figure 1). Information on breed, sex, and age was documented for all sampled dogs. For client-owned dogs, a history of recent ectoparasiticide treatment (within the preceding month) was collected via a standardized owner questionnaire. Approximately 80% of owned dogs had received some form of ectoparasiticide treatment. However, as products were sourced variably (from veterinary clinics or commercial platforms), the specific active ingredients, application dates, and thus the resulting efficacy against ticks were inconsistent and could not be standardized. This potential variability is acknowledged as a factor in the analysis of infestation and infection rates.

Blood specimens were temporarily stored at 4 °C and processed within three days for blood smear preparation and genomic DNA extraction.

Concurrently, 184 ticks were collected from the same cohort of 379 dogs. Ixodid ticks were removed from preferred attachment sites, including the dorsal ear surface, inner pinnae, thoracoabdominal region, limbs, axillae, and inguinal areas. Using forceps, ticks were grasped by the idiosoma or capitulum, as close to the skin as possible and detached by gentle, perpendicular traction to ensure complete removal of the intact mouthpart. Each specimen was individually labeled with its corresponding host and collection site data before transport to the laboratory.

### 2.2. Tick Sample Processing

Initially, ticks were sorted according to sex. To restore mobility, specimens were placed on soft brushes; once active, they were immediately immersed in 80–90 °C water for rapid thermal euthanasia. Subsequently, ticks were rinsed in phosphate-buffered saline (PBS) and vortex-mixed to remove surface contaminants. Each cleaned tick was individually preserved in a 1.5 mL microcentrifuge tube containing 70% ethanol (Xinjiang Watson Biotechnology Co., Ltd., Urumqi, China)for long-term storage.

### 2.3. Morphological Identification of Ticks

Morphological identification was conducted under a stereomicroscope using established taxonomic keys [13]. Key diagnostic structures, including the basis capituli, scutum, spiracular plate, genital aperture, adanal plates, anal groove, and coxae were meticulously examined for species determination. Key morphological features were photographically recorded for documentation and verification.

### 2.4. Tick Colony Rearing

Female ticks collected from canine hosts were classified into three physiological categories: unfed, partially engorged, and fully engorged. Fully and partially engorged specimens were maintained separately in 5 mL centrifuge tubes fitted with perforated caps containing 3–5 ventilation holes. Filter paper strips were inserted into each tube to maintain humidity, and a breathable fabric barrier was placed between the cap and the tube rim. All tubes were maintained in humidity-controlled chambers under optimal temperature conditions.

Approximately seven days after incubation, female ticks oviposited translucent egg masses. Eggs hatched into larvae within 15 days. High-density larval populations were subdivided into multiple tubes to facilitate subsequent experimental procedures. Four 6-month-old New Zealand White rabbits were obtained from the Laboratory Animal Center of Xinjiang Medical University. At the beginning of the experiment, the fur on the dorsal surface of the rabbit’s ears was removed. Approximately 20 larvae were introduced into the dorsal aspect of each ear chamber. Ears were immobilized using restraint devices to prevent self-inflicted injury and maintain chamber integrity [14]. Rabbit health, chamber integrity, and tick attachment were monitored daily. Engorged larvae naturally detached after 5–7 days and were transferred to ventilated microcentrifuge tubes for continued development. Larvae molted into nymphs after approximately 11 days post-detachment. The resulting nymphs were infested onto new rabbit hosts using the same protocol. Fully engorged nymphs detached within approximately 5 days, were collected in perforated microcentrifuge tubes, and subsequently molted into adult ticks after about 15 days under controlled conditions.

### 2.5. DNA Extraction and PCR Amplification

Genomic DNA was extracted from blood and tick samples using the TIANGEN Blood/Cell/Tissue DNA Kit according to the manufacturer’s instructions. Molecular identification of tick species was performed by amplifying the cytochrome c oxidase subunit 1 (CO1) gene [15] and Internal Transcribed Spacer 2 (ITS2), The thermal cycling conditions were adopted from previously published protocols [16].

For pathogen screening, PCR assays targeting *Hepatozoon* spp., *Rickettsia* spp., *Anaplasma* spp., and *Babesia* spp. were conducted using primers synthesized by Sangon Biotech (Shanghai, China), primer sequences are listed in Table 1.

All PCR reactions were performed in a final volume of 13 µL, containing 2 μL template DNA (25–35 ng/μL), 6.5 μL 2× Taq PCR Master Mix (KT201; TIANGEN BIOTECH, Beijing, China), 0.5 μL each of forward and reverse primers (10 μM), and 3.5 μL nuclease-free water. Nuclease-free water served as the negative control. Known positive DNA for each target pathogen was included in all PCR runs as a positive control PCR amplicons were separated by electrophoresis on 1.5% agarose gels [17], stained with 4S Green Plus nucleic acid dye, and visualized under ultraviolet light.

**Table 1:** List of primers for molecular identification and pathogen detection.

Pathogen and Target Gene	Fragment Length (bp)	Primers (5′-3′)	PCR Conditions	References
ITS2	821 bp	TCGTCTGTCTGAGGGTCGGA ATCGTCTCGTGTAGCGTCG	Initial denaturation: 95 °C for 5 min followed by 35 cycles of denaturation at 95 °C for 30 s, annealing at 55 °C for 30 s, extension at 72 °C for 30 s, and final extension at 72 °C for 5 min.	[18]
CO 1	709 bp	GGTCAACAAATCATAAAGATATTGG TAAACTTCAGGGTGACCAAAAAATCA	Initial denaturation: 95 °C for 5 min followed by 35 cycles of denaturation at 95 °C for 30 s, annealing at 54 °C for 30 s, extension at 72 °C for 30 s, and final extension at 72 °C for 5 min.	[19]
*Hepatozoon* spp. (18S rRNA)	666 bp	ATACATGAGCAAAATCTCAAC	Initial denaturation: 95 °C for 5 min followed by 35 cycles of denaturation at 95 °C for 30 s, annealing at 48 °C for 30 s, extension at 72 °C for 40 s, and final extension at 72 °C for 5 min.	[20]
		CTTATTATTCCATGCTGCAG		
*Rickettsia* spp. (OmpA)	632 bp	ATGGCGAATATTTCTCCAAAA	Initial denaturation: 95 °C for 5 min followed by 33 cycles of denaturation at 95 °C for 30 s, annealing at 55 °C for 30 s, extension at 72 °C for 50 s, and final extension at 72 °C for 5 min.	[21]
		GTTCCGTTAATGGCAGCATCT		
*Anaplasma* spp. (groEL)	688 bp	GAAGATGCWGTWGGWTGTACKGC	Initial denaturation: 95 °C for 5 min followed by 30 cycles of denaturation at 95 °C for 30 s, annealing at 54 °C for 30 s, extension at 72 °C for 60 s, and final extension at 72 °C for 5 min.	[22]
		AGMGCTTCWCCTTCWACRTCYTC		
	446 bp	ATTACTCAGAGTGCTTCTCARTG	Initial denaturation: 95 °C for 5 min followed by 30 cycles of denaturation at 95 °C for 30 s, annealing at 57 °C for 30 s, extension at 72 °C for 60 s, and final extension at 72 °C for 5 min.	
		TGCATACCRTCAGTYTTTTCAAC		
*Anaplasma ovis* (Msp4)	852 bp	GGGAGCTCCTATGAATTACAGAGAATTGTTTAC	Initial denaturation: 95 °C for 5 min followed by 35 cycles of denaturation at 95 °C for 30 s, annealing at 62 °C for 30 s, extension at 72 °C for 52 s and final extension at 72 °C for 5 min.	[23]
		CCGATCCTTAGCTGAACAGGAATCTTGC		
*Babesia* spp. (18S rRNA)	643 bp	GTGAAACTGCGAATGGCTCA	Initial denaturation: 95 °C for 5 min followed by 35 cycles of denaturation at 95 °C for 30 s, annealing at 55 °C for 30 s, extension at 72 °C for 90 s and final extension at 72 °C for 5 min.	[24]
		CCATGCTGAAGTATTCAAGAC		

### 2.6. Gel Extraction and Sequencing

Bands corresponding to the expected amplicon size were excised, purified using an agarose gel DNA recovery kit (TIANGEN BIOTECH, Beijing, China), and sequenced. The resulting nucleotide sequences were compared against the NCBI GenBank database using BLAST to determine sequence identity and query coverage [25]. Multiple sequence alignment was performed using the Clustal X program to assess similarity among the obtained isolates and related reference sequences.

### 2.7. Phylogenetic Tree Construction

Phylogenetic analysis was conducted in MEGA 11 [26] by aligning experimental sequences with homologous reference sequences retrieved from the GenBank. Multiple sequence alignment was performed using MUSCLE algorithm [27], followed by manual trimming of ambiguous regions to improve alignment accuracy. Phylogenetic analysis was conducted in MEGA 11 using the Maximum Likelihood method. The best-fit substitution model for each gene was selected based on the lowest Bayesian Information Criterion (BIC) score calculated by ModelFinder within MEGA. The following models were applied: the General Time-Reversible model with Gamma distribution and Invariant sites (GTR + G + I) for *Babesia* spp. and *Anaplasma* spp., the Tamura 3-parameter model with Gamma distribution (T92 + G) for *Rickettsia* spp., and the Hasegawa-Kishino-Yano model with Gamma distribution (HKY + G) for the tick ITS2 analysis. Outgroups were selected based on taxonomic proximity: *Drosophila melanogaster* (Accession No. KCO135421) for tick phylogeny, *Theileria sp*. (Accession No. AF036336) for *Babesia* spp., *Orientia tsutsugamushi* (MH168016) for *Rickettsia* spp., *Ehrlichia canis* (AB287435) for *Anaplasma* spp. Maximum-likelihood phylogenetic trees were constructed under the Tamura-Nei model with 1000 bootstrap replicates to assess nodal support.

### 2.8. One-Way Analysis of Variance (ANOVA)

To ensure robust statistical interpretation of the epidemiological data, the analysis was tailored to sample size. For regions with sufficient sample sizes (*n* ≥ 10), comparative statistical analyses were conducted. The 95% confidence intervals for pathogen prevalence rates were calculated using the Wilson score interval method. Differences in detection rates between major regions, as well as among different pathogens, were assessed using Fisher’s exact test or chi-square test, as appropriate for categorical data. For regions with limited sample sizes (*n* ≤ 10), only descriptive statistics (prevalence with 95% confidence intervals) are reported, and formal comparative tests (including one-way ANOVA) were omitted. Consequently, inter-regional statistical comparisons were restricted to areas with adequate sample sizes, such as Urumqi, Changji, and Hotan. All statistical analyses were performed using GraphPad Prism 6.0.

## 3. Results

### 3.1. Morphological Examination and Colony Development of Ticks

**Figure 2:**
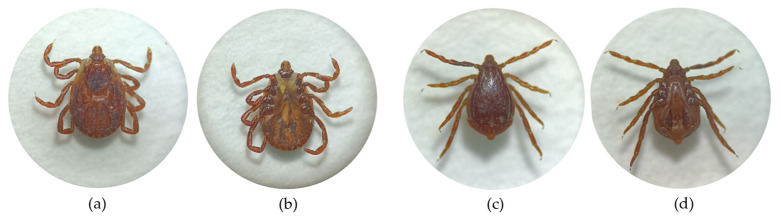
(**a**) Dorsal view of female *Rh. turanicus* (2 × 10); (**b**) ventral view of female *Rh. turanicus* (2 × 10); (**c**) dorsal view of male *Rh. turanicus* (2.5 × 10); (**d**) ventral view of male *Rh. turanicus* (2.5 × 10).

A total of 184 ticks were collected from the dogs. All ticks were morphologically identified as *Rh. turanicus*, comprising 184 adults (140 females, 44 males). The collected ticks were morphologically examined under a stereomicroscope, with representative specimens shown in Figure 2.

**Figure 3:**
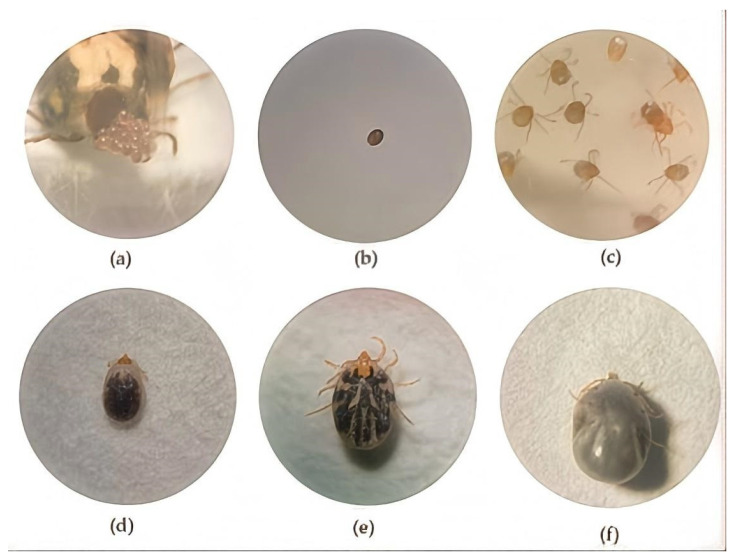
Representative developmental stages of *Rh. turanicus* observed under stereomicroscopy: (**a**) ovipositing adult female tick (*Rh. turanicus* 3.0 × 10); (**b**) tick egg (*Rh. turanicus* 3.0 × 10); (**c**) larval stage (unfed) (*Rh. turanicus* 3.0 × 10); (**d**) engorged larva (*Rh. turanicus* 3.0 × 10); (**e**) unfed nymph (*Rh. turanicus*, 3.0 × 10); (**f**) engorged nymph (*Rh. turanicus*, 3.0 × 10).

Engorged ticks were maintained on experimental rabbits to observe the completion of their life cycles. As depicted in Figure 3, each developmental stage (larva, nymph, adult) required a blood meal, after which ticks detached spontaneously upon engorgement. The estimated developmental period from egg to adult was 50 days, indicating a rapid lifecycle and substantial reproductive potential, a characteristic of significant epidemiological importance.

### 3.2. PCR Detection of Tick-Borne Pathogens in Canine Blood

In the present study, molecular screening for *Anaplasma* spp., *Hepatozoon* spp., *Rickettsia* spp., and *Babesia* spp. was performed on canine blood-derived DNA. The prevalence of these pathogens was determined to be *Anaplasma* spp., *Rickettsia* spp. and *Babesia* spp. was 54/379 (14.25%) for *Anaplasma* spp., 10/379 (2.64%) for *Hepatozoon* spp., 82/379 (21.64%) for *Rickettsia* spp., and 83/379 (21.90%) for *Babesia* spp. (As shown in Table 2).

### 3.3. Molecular Screening of Tick Samples and Phylogenetic Analysis

Ticks are key arthropod vectors capable of transmitting a wide range of viral, bacterial, rickettsial, and protozoan pathogens to both humans and animals [28,29]. Their global distribution and capacity to harbor multiple infectious agents underscore their profound public health significance. Molecular screening of 184 tick samples yielded the following detection rates: *Anaplasma ovis* was identified in 28/184 (15.22%) specimens, detected via 852 bp *msp*4 gene amplification; *Hepatozoon* spp.: 15/184 (8.15%), determined by 666 bp 18S rRNA fragment, and *Rickettsia* spp.: 40/184 (21.74%), confirmed by targeting the 652 bp *omp*A gene. (As shown in Table 3).

**Figure 4:**
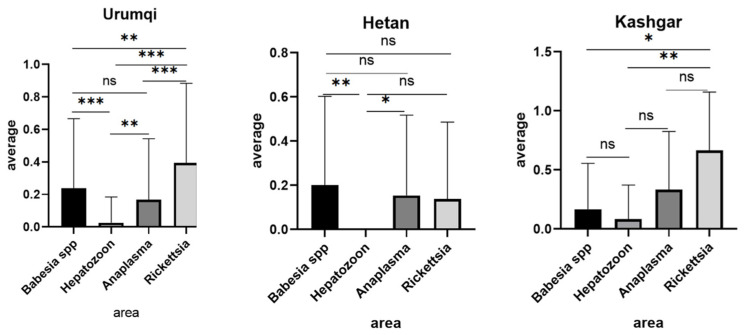
Comparative analysis of pathogen infection rates across selected regions.(* *p* < 0.05, ** *p* < 0.01, *** *p* < 0.001, ns (not significant)).

To evaluate the prevalence of the four pathogens across different regions, one-way analysis of variance (ANOVA) was performed. As shown in Figure 4, samples from Urumqi exhibited statistically significant or highly significant differences (*p* < 0.01) in infection rates among most detected pathogens. However, no significant difference was observed between *Babesia* spp. and *Anaplasma* spp. infections (*p* > 0.05). Statistical comparisons for other regions were omitted due to insufficient sample sizes, which limited the reliability of interregional comparisons.

### 3.4. Phylogenetic Analysis

**Figure 5:**
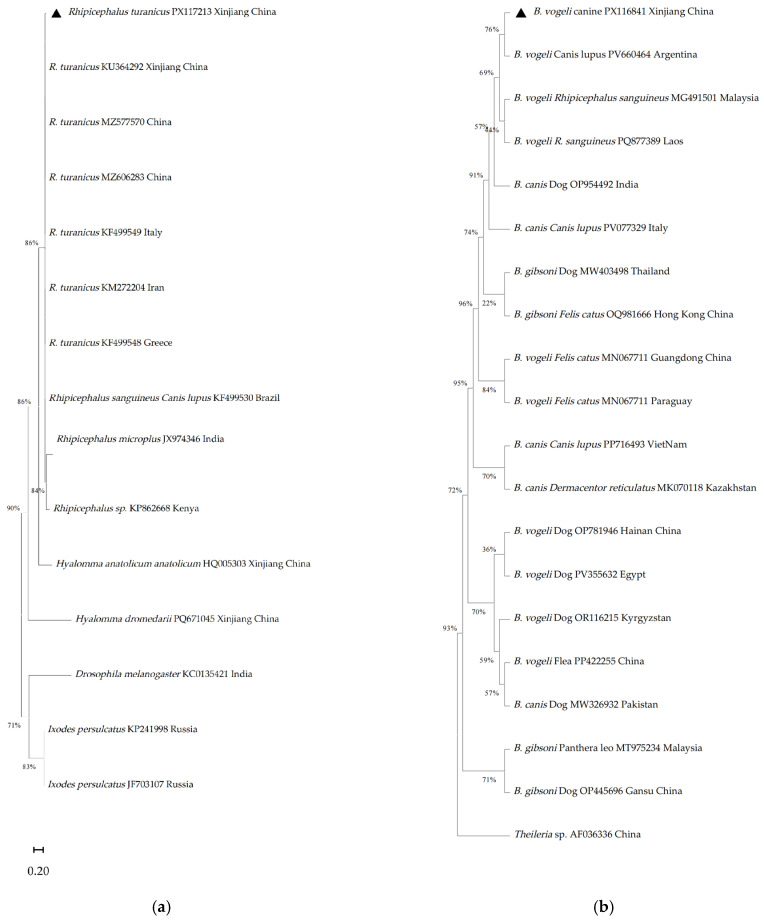

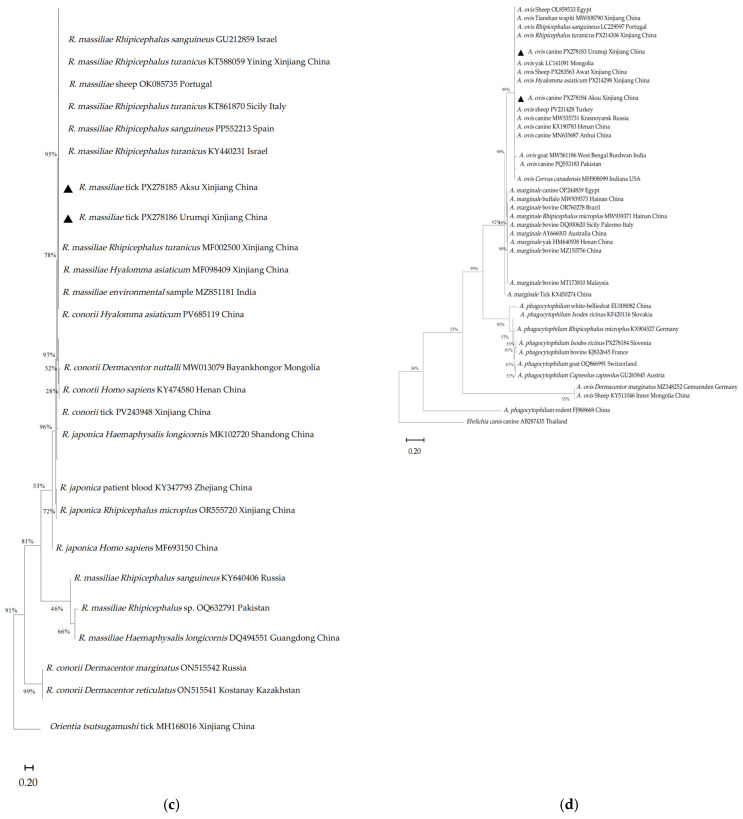
Phylogenetic analysis of tick species and associated pathogens. (**a**) Phylogenetic relationship of ticks; (**b**) phylogenetic relationships of the genus *Babesia*; (**c**) phylogenetic relationships of the genus *Rickettsia*; (**d**) phylogenetic relationships of the genus *Anaplasma*.(The sequences presented in black triangle represent the sequences obtained in this article).

BLASTn analysis revealed high sequence identity for all major targets: the *Rh. turanicus* ITS2 sequence (PX117213) showed 99.30% identity with a *Rh. turanicus* reference from China (MZ536629); the Anaplasma Msp4 sequence (PX278184) showed 100% identity with *A. ovis* from Egypt (OP244841); the Babesia 18S rRNA sequence (PX116841) showed 99.18% identity with *Babesia vogeli* from India (OP954492); and the Rickettsia OmpA sequence (PX278185) showed 99.18% identity with *Rickettsia massiliae* from Lebanon (KY233237. Phylogenetic relationships among the tick species identified in this study and detected pathogens (*Babesia* spp., *Hepatozoon* spp., and *Anaplasma* spp.), together with their corresponding reference sequences, are presented in Figure 5.

Sequences generated in this study are denoted by a triangle symbol preceding their respective accession numbers. Internal transcribed spacer 2 (ITS-2) rDNA sequences, each 832 bp in length, were successfully amplified from individual tick amplicons. Phylogenetic comparisons revealed that the representative tick sequence from Xinjiang, China (Accession No. PX117213), BLASTn analysis revealed high nucleotide identity for all major targets: the *Rh. turanicus* ITS2 sequence (PX117213) showed 99.30% identity with a reference from China (MZ536629) [30,31]; the *Anaplasma* spp. Msp4 sequence (PX278184) showed 100% identity with *A. ovis* from Egypt (OP244841); the *Babesia* spp. 18S rRNA sequence (PX116841) showed 99.18% identity with *B. vogeli* from India (OP954492); and the *Rickettsia* spp. OmpA sequence (PX278185) showed 99.18% identity with *R. massiliae* from Lebanon (KY233237). In the phylogenetic trees, our sequences clustered within clades containing reference strains from geographically diverse regions, supported by bootstrap values > 70% at key nodes. The *Babesia* spp. sequence grouped with *B. vogeli* strains from Argentina, Malaysia and Laos, while the *Rickettsia* spp. and *Anaplasma* spp. sequences clustered with isolates from China, India, Portugal and Central Asia, indicating genetic links to global lineages, suggesting regional circulation with transboundary genetic links.

## 4. Discussion

Pet ownership is particularly prevalent among urban young adults in China, with statistical evidence indicating higher pet-keeping trends within this demographic [32]. A recent population-based study further stratified the prevalence of pet ownership across different age groups (≤25, 25–34, 35–44, 45–54, and ≥55 years), reporting corresponding rates of 23.9%, 23.4%, 22.5%, 18.2%, and 18.3%, respectively. Pet ownership among the youngest cohort (≤25 years) was significantly higher than that observed in the oldest group (≥55 years) (*p* < 0.05) [33]. Against the backdrop of rapid urbanization and modernization in contemporary China, younger generations (typically defined as individuals aged 18–40) are experiencing lifestyle shifts within a novel socioeconomic context distinct from earlier generations [34]. These demographic patterns suggest an increasing emotional reliance on pets for companionship, potentially driven by changing social dynamics and an aging population. This is further reflected in the increasing penetration rate of pets in Chinese households, underscoring their enhanced social and emotional significance. Consequently, public awareness of and demand for information on pet-associated diseases have increased. Despite this strengthening human–animal bond, tick-borne diseases (TBDs) remain endemic across diverse regions of Xinjiang, China. Tick bites can transmit a various pathogens to dogs, including *Babesia*, *Rickettsia*, *Hepatozoon*, and *Anaplasma* [35]. Geographically, Xinjiang shares borders with eight countries, Mongolia, Russia, Kazakhstan, Kyrgyzstan, Tajikistan, Afghanistan, Pakistan, and India, stretching from its northeast to southwest. Due to this transboundary continuity, the tick species and spectrum of TBDs in Xinjiang are likely comparable to those reported in neighboring nations, thereby providing an important comparative framework for the present investigation.

This study investigated the presence of pathogens in canine blood samples and in ticks collected directly from dogs across multiple locations in Xinjiang, including Urumqi, Changji, Shihezi, Bole, Hotan, Kashgar, Kizilsu Kyrgyz Autonomous Prefecture (hereafter referred to as “Kizilsu”), Wujiaqu, and Aksu. A combined approach integrating stereomicroscopic examination and PCR assays was applied to analyze 379 canine blood samples and their associated tick specimens, revealing notable infection rates for several pathogens.

Discrepancies observed between morphological and molecular identifications of tick species in this study underscore the necessity of adopting an integrative multi-method strategy for identifying complex tick species, particularly cryptic species or species complexes. The short developmental cycle observed in *Rh.* *turanicus* (approximately 50 days under laboratory conditions) suggests a high reproductive capacity. Since a single engorged female can lay thousands of eggs, and each developmental stage (larva, nymph, and adult) requires a blood meal, there are multiple opportunities for pathogen acquisition and transmission. This rapid population turnover may amplify the transmission risk of pathogens such as *Babesia canis* and *Rickettsia* spp., particularly in areas with high tick densities. This conclusion is supported not only by our present data but also by our team’s prior research and other regional studies. For instance, in earlier work from southern Xinjiang, *Rh. turanicus* and *H. anatolicum* were identified as dominant tick species on sheep, with molecular detection revealing high prevalence of pathogens such as *Rickettsia* spp., *Theileria* spp., and *Anaplasma* spp. in both species, underscoring their potential role as key regional vectors [16]. However, the accurate identification of *Rh. turanicus*, a taxonomically debated complex, remains challenging. Several studies indicate that relying solely on morphological traits may be insufficient to reliably distinguish it from closely related congeners, such as *Rh. sanguineus* sensu lato.

*Babesiosis*, a zoonotic arthropod-borne disease caused by intraerythrocytic protozoan parasites of the genus *Babesia* [36], is primarily transmitted through tick bites, although alternative transmission routes, including blood transfusion and aggressive interactions between dogs, have also been documented [37]. In China, the disease is endemic across numerous provinces, including Xinjiang, Sichuan, Shaanxi, Gansu, Zhejiang, Henan, Anhui, Shandong, and Hubei, and typically exhibits seasonal peaks from March to October. Despite this broad distribution, reports from Xinjiang remain sparse, sporadic cases have been reported in other regions, such as Heilongjiang Province [38]. In the current study, *Babesia* spp. was detected in 83 of the 379 canine blood samples (21.90%), yet it remained absent in all collected tick specimens. Across both northern and southern Xinjiang, the prevalence in canine hosts consistently exceeded 20% in several surveyed locations. The failure to detect the pathogen in ticks, despite the high prevalence in dogs, may be attributable to low parasitemia within the vectors, or pathogen clearance following salivary transmission during feeding, potentially reducing detectability using current molecular assays. Consistent with previous reports, the detection of *B. vogeli* in southern China [39] and the identification of *Rh. sanguineus* as the principal vector of canine babesiosis in the Guangzhou region further support the epidemiological relevance of our findings.

Diseases caused by *Rickettsia* species belong to the order *Rickettsiales*. In Pakistan, *Rickettsia* spp. have been identified in several tick species, including *Rh. turanicus*, *Rh. microplus*, *Haemaphysalis* spp., and *Hyalomma asiaticum* [40]. Our study identified a *Rickettsia* detection rate of 21.64% in canine blood samples, with notably higher incidence observed in Urumqi and Aksu. A related investigation from Pakistan reported a slightly lower prevalence (19%), which may be attributable to the shared border between Xinjiang and regions of Pakistan, as well as similar climatic conditions that facilitate tick dispersal across these areas. Additional factors, such as differences in tick sample size, extensive desert landscapes in Pakistan, which may be less conducive to tick survival and reproduction, could also contribute to reduced detection rates. In contrast, in India, the flea *Xenopsylla cheopis*, rather than ticks, has been identified as the primary vector of *Rickettsia typhi*, the causative agent of endemic murine typhus [41], likely due to the flea’s broader environmental adaptability. A comparative study detected *Rickettsia* in *Rh. turanicus* in Kyrgyzstan [42], a species also identified in our survey. The co-detection of *Babesia* spp. infections in canine blood suggests a potential vector role for *Rh. turanicus* in the region. Consistent with these observations, *Rickettsia* spp. has also been reported in multiple tick species in Kazakhstan [43] and documented in a recent study from Pakistan [44].

The overall detection rate for *Anaplasma* spp. was 14.25% (54/379). The pathogen was detected in canine blood samples from Urumqi, Changji, Aksu, and Hotan, but was not identified in samples from Bole, Kashgar, and Kizilsu, this discrepancy is likely due to the smaller sample sizes obtained from the latter regions. Notably, a 100% infection rate (4/4) was observed in samples collected from Aksu. A previous investigation in Kyrgyzstan, which analyzed 494 engorged ticks collected from multiple host species (including cattle, horses, sheep, chickens, dogs, and cats) across six northern districts, identified 34 ticks collected from dogs as *Rh. turanicus*, a species also detected in the present study, but did not detect Anaplasma [45]. Conversely, another report confirms *Anaplasma* spp. infections in cattle, horses, dogs, and cats in Kyrgyzstan [46]. Given that Kyrgyzstan shares borders with Kizilsu, Kashgar, and Aksu prefectures in Xinjiang, a higher infection rate was observed in our study, compared to the recent Kyrgyz study [45]. It might be attributed to pronounced regional climatic heterogeneity within Kyrgyzstan, which can substantially influence tick distribution and pathogen prevalence. Similarly, a study from Mongolia reported the detection of *Anaplasma platys* in only 1% (1/100) of dog blood samples, while other data indicate a significantly higher prevalence of *Anaplasma* spp. (57.6%) [47]. The comparatively low prevalence reported in the former study may be explained by the absence of competent vector ticks, such as *Rhipicephalus* spp., in certain regions of Mongolia, or by enhanced innate or acquired immunity in more active, free-roaming Mongolian dogs [48].

*Canine hepatozoonosis*, principally caused by *Hepatozoon canis* and *H. americanum*, is a ubiquitous TBD reported worldwide. These pathogens exhibit distinct tissue tropism: *H. canis* predominantly infects the spleen, bone marrow, and lymph nodes, whereas *H. americanum* primarily targets skeletal and cardiac muscle. Hepatozoonosis affects domestic dogs, felids, and wildlife, including a documented case in a wild jackal in India [49]. The tick *Rhipicephalus sanguineus* sensu lato (s.l.) serves as a confirmed or potential vector for babesiosis, ehrlichiosis, and hepatozoonosis, frequently facilitating mixed parasitic infections in dogs [50]. Beyond tick-mediated transmission, a study conducted near Mazar-i-Sharif in northern Afghanistan, which screened 751 rodents and other small mammals for apicomplexan parasites using quantitative PCR (qPCR) on kidney and spleen tissues, detected *Hepatozoon*-positive house mice at all sampling sites, with significant variation in positivity rates among the three surveyed locations [5]. These findings suggest that, in addition to ticks, rodents and other wildlife may contribute to the maintenance and transmission of *Hepatozoon*, potentially acting as reservoirs or intermediate hosts for ticks in southwestern Xinjiang, particularly in areas such as Kashgar near the Pamir Plateau. Pathogen surveillance studies in ticks from northeastern China detected *Hepatozoon* spp. in multiple tick species, including *Dermacentor nuttalli*, *D. silvarum, H. concinna, H. longicornis*, and *I. persulcatus* [9], but not in the *Rhipicephalus species* identified in our study, suggesting regional vector diversity. This observation is corroborated by reports from Brazil, where *Amblyomma ovale*, *Amblyomma cajennense*, and *Rh. microplus* are considered competent vectors alongside *Rh. sanguineus* [51]. Furthermore, research in Shaanxi, China, demonstrated that the severity of clinical signs in *canine hepatozoonosis* correlates with the degree of parasitemia and identified *H. canis* in *Haemaphysalis longicornis* [52]. In our study, *Hepatozoon* spp. were found in 10 of 379 canine blood samples (2.64%) and in 15 of 184 tick samples (8.15%). The pathogen was detected in dog blood only from Urumqi, Changji, and Kashgar, whereas ticks carrying the pathogen were also recovered from Aksu. This discrepancy could arise if ticks harboring the pathogen may not yet have transmitted it to their canine hosts at the time of sampling, particularly given that infection can occur through the ingestion of infected ticks (e.g., *Rh. sanguineus*) [53]. Our findings suggest that the prevalence of this pathogen in Xinjiang mirrors broader regional trends, likely influenced by shared borders, ecological conditions, and vector distributions. For example, the infection rates of *Hepatozoon* spp. reported in Chongqing and Hubei Provinces were 3.27% [53]; however, data specific to Xinjiang remain limited, underscoring the need for expanded regional surveillance.

Molecular analysis confirmed the identity of the detected pathogens and their genetic relationships to global strains. BLASTn alignment revealed high nucleotide identity between our sequences and established reference strains: 99.30% for *Rh. turanicus* (ITS2), 100% for *A. ovis* (Msp4), 99.18% for *B. vogeli* (18S rRNA), and 99.18% for *R. massiliae* (OmpA). These results robustly support the species-level designation of the pathogens circulating in Xinjiang’s canine population. Subsequent phylogenetic reconstruction provided insights into their evolutionary context. While high BLAST identity anchored the classifications, the phylogenetic trees occasionally showed modest bootstrap support at deeper nodes, and the placement of some reference sequences (e.g., certain *Rh. turanicus* and Babesia references) did not strictly correlate with geographic origin. This discordance between high sequence similarity and variable phylogenetic resolution, particularly for the Babesia 18S rRNA and tick ITS2 markers, may reflect the limitations of single-gene phylogenies for closely related taxa or indicate the presence of conserved genomic regions alongside more variable ones that influence tree topology [54]. Notably, the clustering of our *A. ovis* and *R. massiliae* sequences with isolates from distant regions (Egypt, Lebanon) suggests potential long-range dispersal pathways, possibly linked to animal movement or widely distributed vector species. Conversely, the high homology of our *B. vogeli* sequence with strains from neighboring India aligns with expected regional transmission patterns across permeable borders. This study underscores that while core genetic identity is conserved, the phylogenetic signals for some pathogens in Xinjiang are complex, potentially shaped by regional host–parasite dynamics, ancestral polymorphism, or the ongoing introduction of foreign strains. Future studies employing multi-locus or genomic approaches will be crucial to resolve these finer-scale evolutionary relationships and transmission networks.

This identification uncertainty carries direct epidemiological significance. For example, studies have detected *Rickettsia massiliae*, which can be transovarially transmitted, in ticks morphologically identified *Rh. turanicus* [54]. Another survey in Kazakhstan showed that *Rh. turanicus* is one of the carriers of rickettsial pathogens (e.g., *R. raoultii*) [55]. A study in the Junggar Basin reported that ticks collected from pet dogs and identified as *Rh. turanicus* sensu stricto, through combined morphological and molecular methods were found to harbor several important zoonotic pathogens, including *Ehrlichia chaffeensis* and *Anaplasma phagocytophilum* [43]. This collective evidence indicates that, regardless of its precise final taxonomic status, ticks within the *Rh. turanicus* complex play a key role in the maintenance and transmission networks of pathogens in Xinjiang and surrounding areas. Moreover, the host range of these ticks may be wider than previously recognized; for instance, records show that *Rh. turanicus* can also parasitize atypical hosts like lizards [56], increasing the complexity of their dispersal and transmission cycles.

To enable a more accurate assessment of vector potential and disease risk, future surveillance studies should incorporate more than one method for identification. We recommend adopting a comprehensive identification framework that integrates key morphological characteristics, multi-locus (e.g., COI, 16S rRNA, ITS2) phylogenetic analysis, and ecological and geographical distribution data. This study strictly adhered to this principle: when single-gene sequence identity fell below 95% or phylogenetic node bootstrap support was below 70%, we conservatively reported identification results at the genus or species complex level, avoiding overinterpretation of species identity. This cautious approach aims to enhance data reliability and establish a more solid and accurate foundation for understanding the true transmission ecology of Rhipicephalus ticks and their carried pathogens in Xinjiang, a critical region with significant ecological diversity and extensive international borders.

In summary, the simultaneous detection of tick-borne pathogens in canine hosts and ticks highlights the presence of a diverse range of well-characterized and emerging bacterial agents in Xinjiang with zoonotic potential. Consequently, it is crucial to conduct more extensive surveillance to document these pathogens and elucidate their ecological and pathogenic roles in natural environments.

## 5. Conclusions

This study provides a comprehensive assessment of the prevalence and diversity of TBDs in dogs and their associated ticks across selected regions of Xinjiang. The tick species infesting dogs in this study was identified as *Rh. turanicus*. The detection of *Rickettsia* spp., *Anaplasma* spp. (e.g., *A. bovis*), and *Babesia* spp. (e.g., *B. vogeli*) underscores potential public health and veterinary risks, necessitating vigilance from veterinary and public health officials. These findings contribute valuable baseline data to support the development and implementation of region-specific strategies for the prevention and control of TBDs. Nevertheless, this study has limitations related to sampling period, geographic coverage, and host range, as sampling was restricted to canine populations. These constraints may have influenced the representativeness of the data set. Accordingly, sustained, large-scale, and multi-host surveillance programs are urgently needed to more accurately characterize the epidemiology of TBDs in the region. Furthermore, implementation of an integrated One Health approach is essential to advance understanding of the transmission dynamics, ecological drivers, and pathogenic potential of TBDs in China. Adoption of a “One Health” framework is particularly vital for formulating effective integrated pest management strategies to control tick infestations and mitigate the burden of associated TBDs.

## Figures and Tables

**Table 2:** Molecular detection rates of tick-borne pathogens in canine blood samples from different regions.

Area	No. of Blood Samples	No. of Infections (%; 95%CI)			
		*Anaplasma* spp.	*Hepatozoon* spp.	*Rickettsia* spp.	*Babesia* spp.
Urumqi	155	26 (16.7; 10.83–22.72)	4 (2.5; 0.06–5.11)	61 (38.71; 31.58–47.13)	37 (23.87 17.08–30.66)
Changji	122	14 (11.48; 5.60–16.81)	5 (4.1; 0.52–7.48)	5 (4.1; 0.52–7.54)	29 (23.77; 15.70–30.70)
Shihezi	9	-	-	-	-
Bole	13	-	-	-	2 (22.22; 7.31–38.08)
Wuqia	1	-	-	1 (100)	-
Kashi	5	-	1 (20)	2 (40)	-
Aksu	4	4 (100)	-	4 (100)	2 (50)
Hotan	65	10 (15.38; 6.38–24.39)	-	9 (13.85; 5.22–22.47)	13 (20; 10.01–29.99)
Aktao	1	-	-	-	-
Atushi	1	-	-	-	-
Wujiaqu	3	-	-	-	-
Total	379	54 (14.25; 10.71–17.78)	10 (2.64; 1.02–4.26)	82 (21.64; 17.77–26.15)	83 (21.90; 17.72–26.08)

“-“ mean not detected.

**Table 3:** Detection rates of pathogens in tick samples from different regions.

Area	No. of Ticks	No. of Infections (%; 95%CI)			
		*Anaplasma* spp.	*Hepatozoon* spp.	*Rickettsia* spp.	*Babesia* spp.
Urumqi	107	6 (5.6%; 1.18–10.04)	6 (5.61%; 1.18–10.04)	25 (19.69%; 15.22–31.51)	-
Changji	4	4 (100%)	-	-	-
Shihezi	23	-	-	11 (47.83%; 25.74–69.91)	-
Bole	-	-	-	-	-
Wuqia	-	-	-	-	-
Kashi	10	3 (30%; 4.56–64.56)	2 (20%; 10.16–50.16)	-	-
Aksu	22	12 (54.55%; 31.95–77.14)	2 (9.09%; 3.96–22.14)	3 (13.64%; 1.94–29.21)	-
Hotan	8	-	-	1 (12.5%)	-
Aktao	3	1 (33.33%)	-	-	-
Atushi	5	1 (20%)	4 (80%)	-	-
Wujiaqu	2	1 (50%)	1 (50%)	-	-
Total	184	28 (15.21%; 9.98–20.46)	15 (8.15%; 4.16–12.14)	40 (21.74; 15.72–27.74)	-

“-“ mean not detected.

## Data Availability

Data associated with the current study are available from the corresponding author upon reasonable request.
